# Electrical Chorea

**Published:** 1846-03

**Authors:** 


*Electrical Chorea.*—By this title, M. Dubini describes a disease which he has met with thirty-six times, and of which he has not been able to find any account in works. Its principal phenomena consist of strong shocks, succeeding each other at determinate intervals, preceded by heat of the skin and an acceleration of the pulse, resembling fever, and which sometimes bring on paralysis of the limbs. The extreme rapidity of these shocks has led M. Dubini to designate the disease electrical chorea. They generally affect one side of the body, and are quite independent of the will. The disease is always accompanied with great depression. The most frequent cause is fright or presence of worms. During the fit speech is lost, although the mind is unaffected; the tongue swells, deglutition is impeded, and an attack of apoplexy often terminates the scene. At the autopsy, the presence of worms in the intestines is discovered; tubercles in the lungs, a serous effusion under the meninges, and an injected condition of the brain itself are also met with. No one of these changes is always met with, so as to be looked upon as the actual cause of the disease. The actual cautery, narcotics, and bleeding have all failed in its treatment. The only medicines which seem to have been of service, are mercurial frictions, preparations of zinc, arnica, and valerian.—*Ibid.*

